# Identification of Six Potentially Long Noncoding RNAs as Biomarkers Involved Competitive Endogenous RNA in Clear Cell Renal Cell Carcinoma

**DOI:** 10.1155/2018/9303486

**Published:** 2018-10-11

**Authors:** Kang Yang, Xiao-fan Lu, Peng-cheng Luo, Jie Zhang

**Affiliations:** ^1^Department of Urology, Renmin Hospital of Wuhan University, Hubei, China; ^2^Research Center of Biostatistics and Computational Pharmacy, China Pharmaceutical University, Nanjing, China; ^3^Department of bioinformatics & Comp Biology, the University of Texas, MD Anderson Cancer Center, USA; ^4^Basic Medical School, Wuhan University, Wuhan 430071, China

## Abstract

*Background*. Clear cell renal cell carcinoma (ccRCC), the most common subtype of renal cell carcinoma (RCC), usually is representative of metastatic heterogeneous neoplasm that links with poor prognosis, but the pathogenesis of ccRCC remains unclear. Currently, numerous evidences prove that long noncoding RNAs (lncRNAs) are considered as competing endogenous RNA (ceRNA) to participate in cellular processes of tumors. Therefore, to investigate the underlying mechanisms of ccRCC, the expression profiles of lncRNAs, miRNAs, and mRNAs were downloaded from the Cancer Genome Atlas (TCGA) database. A total of 1526 differentially expressed lncRNAs (DElncRNAs), 54 DEmiRNAs, and 2352 DEmRNAs were identified. To determine the connection of them, all DElncRNAs were input to the miRcode database. The results indicated that 85 DElncRNAs could connect with 9 DEmiRNAs in relation to our study. Then, databases of TargetScan and miRDB were used to search for targeted genes with reference to DEmiRNAs. The results showed that 203 out of 2352 targeted genes were identified in our TCGA set. Subsequently, ceRNA network was constructed according to Cytoscape and the targeted genes were functionally analyzed to elucidate the mechanisms of DEmRNAs. The results of survival analysis and regression analysis indicated that 6 DElncRNAs named COL18A1-AS1, WT1-AS, LINC00443, TCL6, AL356356.1, and SLC25A5-AS1 were significantly correlative with the clinical traits of ccRCC patients and could be served as predictors for ccRCC. Finally, these findings were validated by quantitative RT-PCR (qRT-PCR). Based on these discoveries, we believe that this identified ceRNA network will provide a novel perspective to elucidate ccRCC pathogenesis.

## 1. Introduction

Renal cell carcinoma (RCC), the 7th most common malignant tumor in male, was estimated at almost 63,000 newly cases and 14,000 deaths occurring in America in 2016[1]. Epidemiological researches reported that more than half of renal cell carcinomas have local invasive tumors and 17% of patients with distant metastasis at the time of diagnosis [2]. Clear cell renal cell carcinoma (ccRCC), the most frequent RCC subtype, accounts for approximately 90% of RCC [3]. Recently, a series of targeted therapies have improved ccRCCs median overall survival rate, while lots of patients ultimately become resistant to these advances due to the limited effects [4]. Although several evidences have documented that somatic genetic mutations were linked with the formation and progress of ccRCC [5–7], it is imperative to identify more effective biomarkers that could help us uncover more thorough pathogenesis regarding the biological characteristics of ccRCC.

With the development of next-generation sequencing technology to human whole genomes, accumulating analyses suggest that genomes encode proteins finally are less than 2% whereas more than 75% are transcribed into noncoding RNAs [8]. Long noncoding RNAs (lncRNAs), longer than 200 nucleotides in length accounting for most of these noncoding RNAs, have been verified as a new class of dramatically role involved in transcriptional and posttranscriptional regulation during various type of cancer progression [9, 10]. In 2011, the competing endogenous RNA (ceRNA) hypothesis proposed by Salmena* et al. *[11], reported that miRNA could suppress targeted gene transcript through complementation with the mRNA sequence at the posttranscriptional level, leading to a variety of RNAs silence. But lncRNA with partial similar miRNA sequence could function as a miRNA endogenous sponge to affect stability of binding with targeting mRNA via shared miRNA response elements, which might exert a significant effect in pathogenesis of cancer. Since then, this hypothesis has been emerged and validated by several experimental studies. Lv* et al. *identified an epithelial-to-mesenchymal transition lncRNA H19, which could sequester miR-29b-3p from regulating targeted gene* DNMT3B *by acting as a ceRNA [12]. Recently, a new LincRNA SNHG14 was upregulated and facilitated ccRCC cell invasion via sponging miR-203 [13]. However, to the best knowledge of the authors, little is known concerning the investigation of ceRNA related ccRCC based on high-throughput detection with large-scale samples.

The Cancer Genome Atlas (TCGA), a public available platform with more than 30 types of cancer from 11,000 patients at least and their clinicopathological information, has been widely applied by large numbers of researches to explore the genetic basis of tumor according to the high-throughput sequencing [14]. In our research, RNA expression profiles between 539 tumor samples and 72 matched normal samples of ccRCC were downloaded from TCGA project. As a result, 1526 aberrantly expressed lncRNAs were identified according soft R, and the miRcode, a transcriptome-wide microRNA target database was then used to screen the hub differential expressed lncRNAs (DElncRNAs) and DEmiRNAs as well as DElncRNAs-DEmiRNAs interactions. Moreover, the DEmiRNA targeted DEmRNAs and DEmiRNAs-DEmRNAs connections were screened performing with the two popular databases. CeRNA network was then constructed to elucidate the crosstalk of the hub DElncRNAs, DEmiRNAs, and DEmRNAs. Survival analysis and regression analysis were utilized to analyze the correlation between clinical traits of patients and related lncRNA. Finally, these bioinformatics discoveries were validated by quantitative RT-PCR (qRT-PCR).

## 2. Materials and Methods

### 2.1. Acquisition of Clinical Characteristics and RNAseq Data

A total of 539 ccRCC patient information were obtained from TCGA database. After excluding patients without entire follow-up information, 530 patients remained available for survival analysis. Besides, lncRNA, miRNA and mRNA expression files included tumor and corresponding normal samples were also downloaded from TCGA portal (https://cancergenome.nih.gov). Moreover, 30 frozen tissue specimens contained 15 tumor tissues and 15 matched adjacent nontumor tissues were obtained from ccRCC patients in Renmin Hospital of Wuhan University. All tissues were collected immediately after surgical resection, and snap-frozen in liquid nitrogen until RNA extraction. This study was approved by the ethics committee of Renmin Hospital of Wuhan University.

### 2.2. Data Procession of RNAseq

An EdgeR (empirical analysis of digital gene expression data in R) package [15], a package for examining differential expression of replicated count data based on Poisson mode, was used to identify the RNAseq and miRNAseq data of ccRCC. Only the differential expression genes (DEGs) with adj.P.Val < 0.05 and |log_2_ fold change (FC)| ≥ 2 were considered as significant.

### 2.3. Construction of lncRNA-miRNA-mRNA ceRNA Network

The ceRNA network was constructed according to the relationship between lncRNA, miRNA and mRNA. Firstly, aberrantly expressed lncRNA, miRNA, and mRNA were identified, and then lncRNA-miRNA connections were predicted by miRcode [16] (http://www.mircode.org/), a database with more than 10,000 lncRNAs which offers whole transcriptome human miRNA target predictions at the basis of comprehensive GENCODE (http://www.gencodegenes.org/) gene annotation. Subsequently, two famous online databases for miRNA targeted mRNA prediction and functional annotation, TargetScan [17] (http://www.targetscan.org/) and miRDB [18] (http://www.mirdb.org/), were used to predict the miRNA-mRNA interactions. Each targeted mRNA in the present research was then selected to intersect with DEmRNAs. Finally, Cytoscape software [19] (version 3.5.1) was constructed the ceRNA network.

### 2.4. Functional Enrichment Analysis of Targeted DEmRNAs

Database for annotation, visualization, and integrated discovery (DAVID), an online tool, has been used as functional enrichment analysis in many studies. In this paper, DAVID was used for gene ontology (GO) analysis to understand the functions of targeted genes in terms of biological process (BP), cellular component (CC), and molecular function (MF). In addition, Kyoto Encyclopedia of Genes and Genomes (KEGG) and an R Package, clusterProfiler [20], were distinguished pathway enrichment of each targeted gene. Cutoff value was set as* P* value < 0.05.

### 2.5. Quantitative Real-Time PCR (qRT-PCR)

Total RNA from specimens were extracted using TRIzol reagent (Invitrogen, Paisley, UK) and then reverse transcribed to cDNA by performing reverse transcription enzyme system (Thermo Fisher Scientific, Waltham, USA) according to the manufacturer instructions. RT-qPCR was utilized to detect selected lncRNA expression performing with SYBR Green qRT-PCR Master Mix (TaKaRa, Shiga, Japan). 2−ΔΔCt method was used to calculate the results of each lncRNA expression. All primer sequences are listed in Table 1, and glyceraldehyde 3-phosphate dehydrogenase (GAPDH) is designed as an internal control to normalize the lncRNA expression levels.

### 2.6. Statistical Analysis

All results in this paper were expressed as the mean ± SD. The significance of the differences between tumor and normal group was performed with Student's t-test, and the value of *P* < 0.05 was considered significantly. The relationship between dysregulated lncRNAs expression level and patient's survival was evaluated by univariate and multivariate Cox regression analysis. In the predictive model, a formula was calculated to predict survival in the training set as previously described [21, 22]. Each patient was assigned a risk score in the light of the Cox regression coefficients of significant survival lncRNAs, and divided into low-risk or high-risk groups based on the median risk score. The Kaplan-Meier (KM) method was used to estimate overall survival, and Receiver operating characteristic (ROC) analysis was performed to compare the sensitivity and specificity of survival prediction by R Soft (version 3.4.3) [23].

## 3. Results

### 3.1. Identification of DElncRNAs, DEmiRNAs, and DEmRNAs in ccRCC Patients

**Figure 1:**
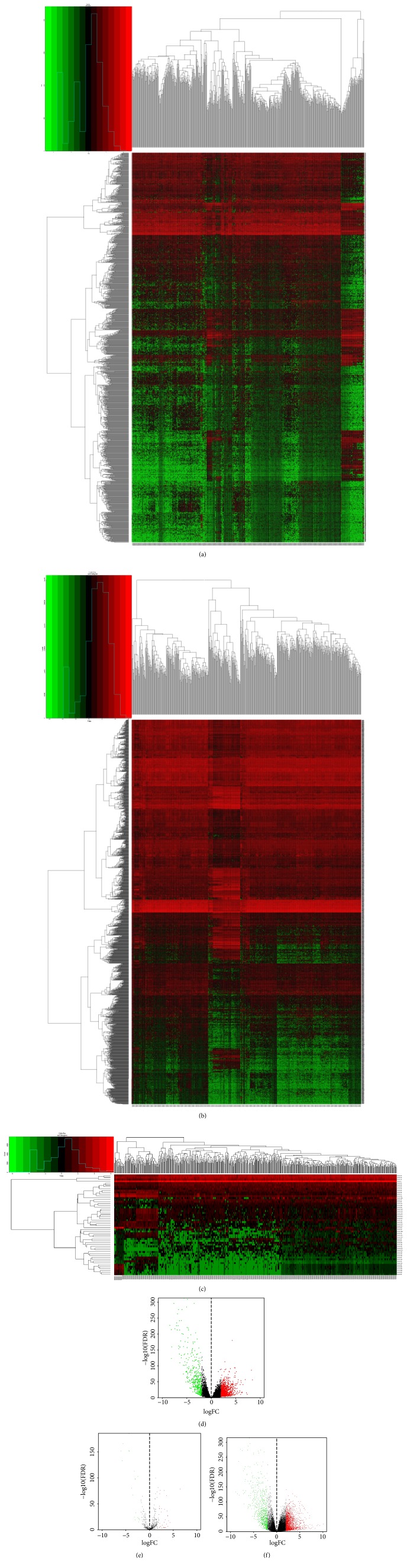
Differential expressed analysis of RNAs from ccRCC patients compared with normal samples. DERNAs were hierarchically clustered by R software (a, b, c). The left longitudinal axis indicated the cluster analysis of DERNAs, and the right axis denoted the results of DElncRNAs, DElncRNAs and DElncRNAs (a-c), respectively. The upper horizontal axis denoted the cluster analysis of each sample, and the down axis below the map corresponded to the results. Each RNA from microarray analysis was plotted into the volcano map, and red color represented the upregulated DElncRNAs, DEmRNAs, and DEmiRNAs (c-e) with log FC ≥ 2 while green represented the downregulated differential expressed RNAs with log_2_ FC < 2. FC, fold change; DE, differential expressed.

To determine the lncRNA, miRNA, and mRNA expression levels in ccRCC, 539 ccRCC patients and 72 normal samples were downloaded from TCGA project. Compared with normal patients, there were 1526 DElncRNAs, 54 DEmiRNAs, and 2352 DEmRNAs, respectively, among which 1083 lncRNAs, 33 miRNAs, and 1599 mRNAs were upexpressed as well as 443 lncRNAs, 21 miRNAs, and 753 mRNAs were downexpressed. To better distinguish the features of these RNAs, clustering analysis and volcano plots were constructed between tumor group and normal group (Figure 1). Each heatmap (Figures 1(a)–1(c)) and volcano plot (Figures 1(d) and 1(e)) showed a distinct separation of distribution.

### 3.2. Prediction of DEmiRNA Targeting DElncRNAs and DEmRNA and Construction of ceRNA Network

**Figure 2:**
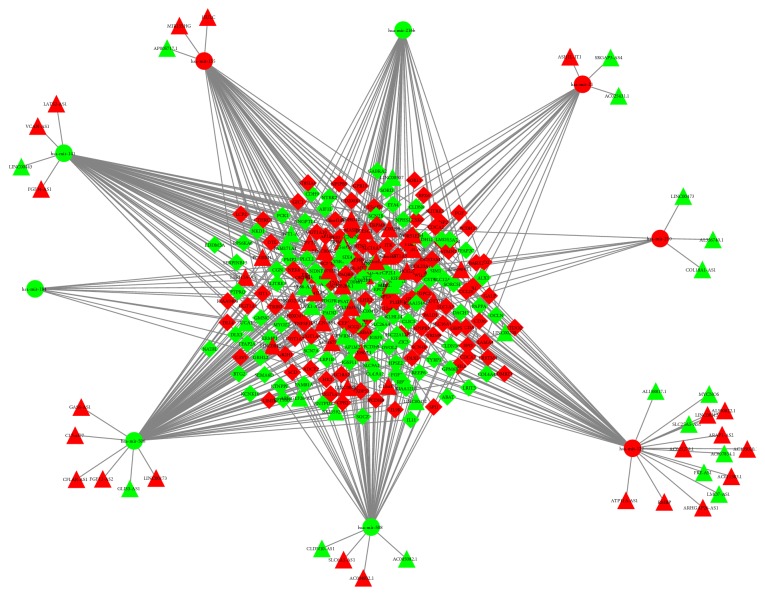
Competitive endogenous RNA network for differential expressed lncRNAs (DElncNNAs), DEmiRNAs, and DEmRNAs in ccRCC. Triangle, ellipse, and diamond denoted DElncRNA, DEmiRNA, and DEmRNA, respectively. Red color represented the upregulated differential expressed RNAs while green represented the downregulated differential expressed RNAs.

To clarify the correlation between miRNA targeting lncRNAs in the ceRNA crosstalk, all DElncRNAs were input to the miRcode database. The results indicated that 85 DElncRNAs could interact with 86 miRNAs. However, only 9 out of 86 miRNAs were discerned in relation to our study (Table S1). We then performed with the databases of TargetScan and miRDB to search for targeted genes with reference to DEmiRNAs. The results showed that 203 out of 2352 targeted genes were identified in the TCGA set (Fig S1). Based on the data as shown above, Cytoscape software was applied to construct and plot lncRNA-miRNA-mRNA ceRNA crosstalk. A total of 85 lncRNAs, 9 miRNAs, and 203 mRNAs were involved in the network (Figure 2).

### 3.3. GO and KEGG Enrichment Analysis of Targeted mRNAs in ceRNA

**Figure 3:**
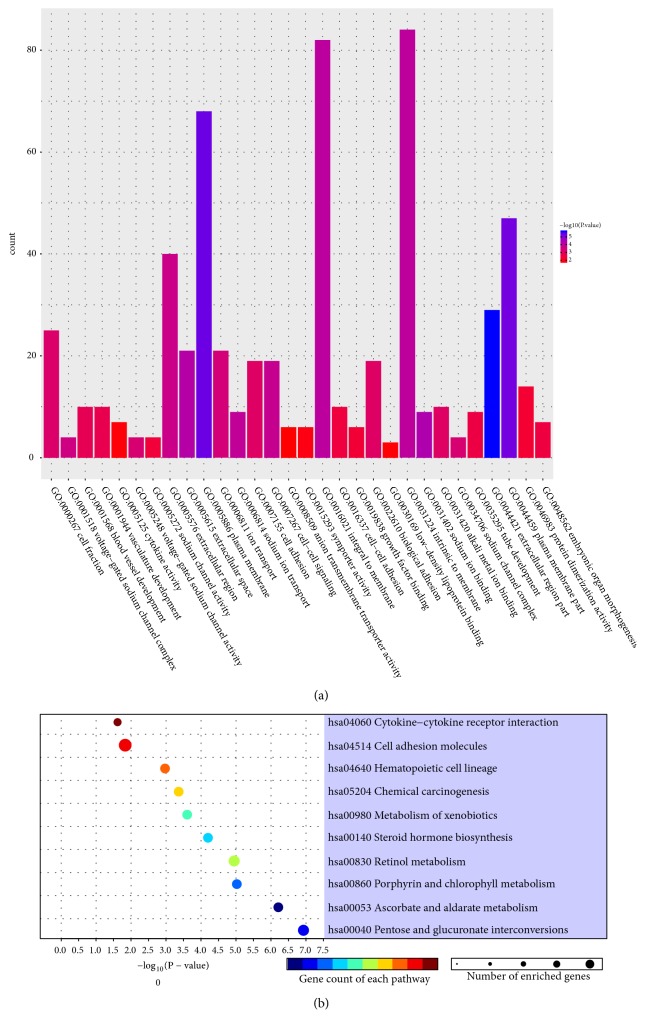
Significant functional analysis and KEGG pathway analysis of the differential expressed mRNAs in ceRNA network. (a) Representative of top 10 GO terms enriched analysis on targeted mRNAs. (b) Representative of top 10 enriched pathways with* P* value less than 0.05. KEGG, Kyoto Encyclopedia of Genes and Genomes; ceRNA, competitive endogenous RNA; GO, gene ontology.

To better comprehend the overall biological features and the signal pathways in relation to the ceRNA network, these mRNAs were investigated by DAVID database and a package, clusterProfiler in R software. As shown in Table 2 and Figure 3(a), these mRNAs mainly were enriched in sodium ion transport, cell-cell signaling, and ion transport about BP, and CC terms related to extracellular region part, plasma membrane, and plasma membrane part, as well as MF terms linked to sodium ion binding, voltage-gated sodium channel activity, and alkali metal ion binding. Pathway enrichment analysis suggested that these mRNAs were predominantly enriched in metabolic related pathway, cytokine-cytokine receptor interaction, and other important pathways (Figure 3(b)).

### 3.4. Evaluation of the Significant lncRNAs in ceRNA concerning ccRCC Patients

**Figure 4:**
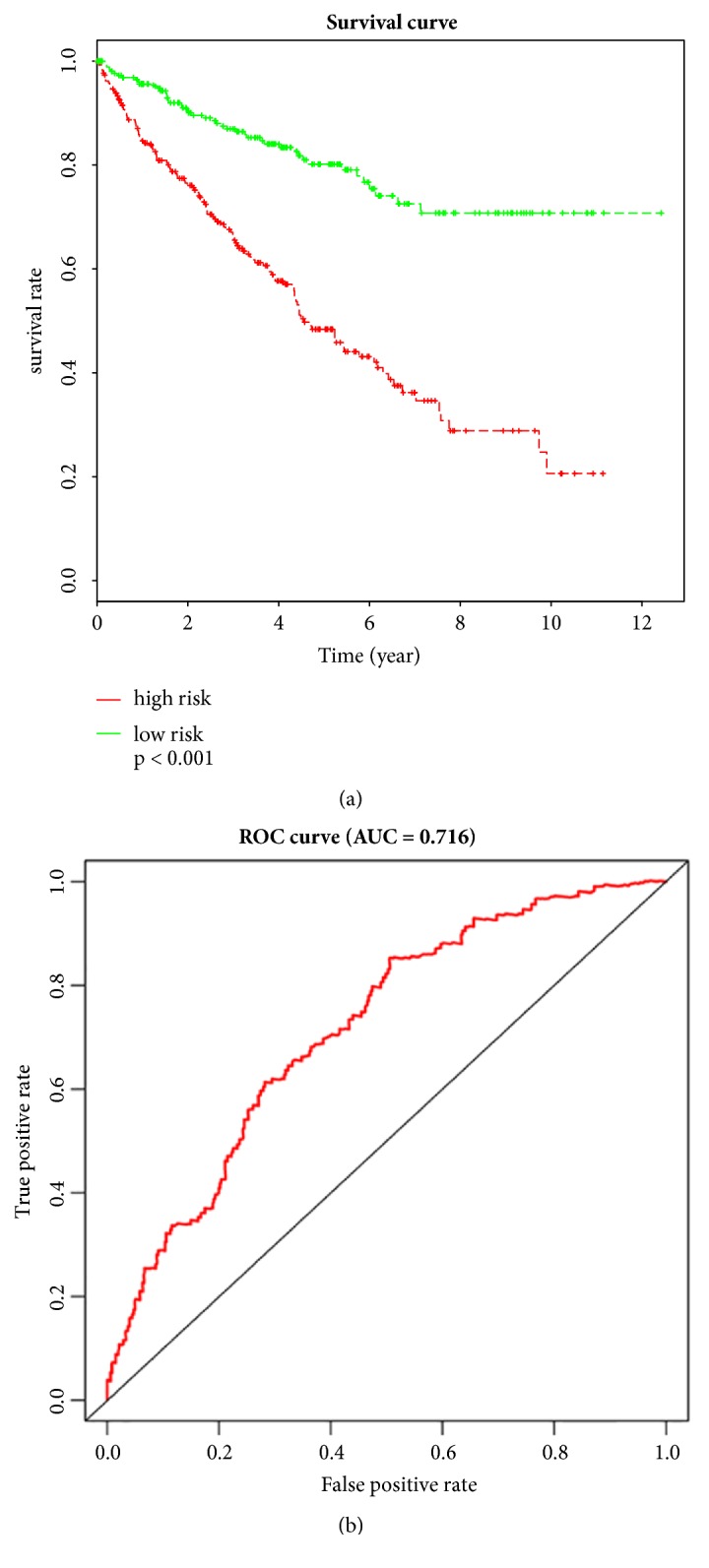
Kaplan-Meier (KM) method and receiver operating characteristic (ROC) analysis of the sensitivity and specificity for overall survival predicted by the six lncRNA risk score. (a) KM plot for a discriminative median patient risk scores with six lncRNA expressions about overall survival.* P* value was calculated by the log-rank test in R software. (b) An ROC curve was built on a univariate regression model based on patient risk scores. The score performance of ROC was estimated by calculating the area under the ROC.

To determine whether those lncRNAs were correlative with the survival of ccRCC patients, we used KM method and univariate Cox proportional hazard regression analysis to test and screen the important lncRNAs linked with survival of the ccRCC patients in the R software. Among 85 aberrantly expressed lncRNAs, 34 lncRNAs with* P* value less than 0.05 were presented by the survival curve (Figure S2). In addition, multivariate Cox regression analysis was carried out to evaluate whether each of these candidates were acted as independent predictors of ccRCC patient's survival. As indicated in Table 3, 6 out of 34 lncRNAs named* COL18A1-AS1, WT1-AS, LINC00443, TCL6, AL356356.1, and SLC25A5-AS1* were served as covariates (*P* < 0.05) in the training set. With the risk score formula as mentioned above, the 6-lncRNA expressed levels were then calculated for each patient in the training set, and patients with risk score were then divided into low-risk groups (n = 265) and high-risk (n = 265) groups via using the median risk score of the test as a cutoff point. The plot results indicated that patients with high-risk had significantly shorter overall survival than those in low-risk group (*P* < 0.001, Figure 4(a)), and ROC analysis revealed that area under receiver operating characteristic (AUC) curve of 6 significant lncRNAs was 0.716 (Figure 4(b)), suggesting that these lncRNAs might be functionally important in pathogenesis of ccRCC patients.

### 3.5. Validation of lncRNAs through qRT-PCR

**Figure 5:**
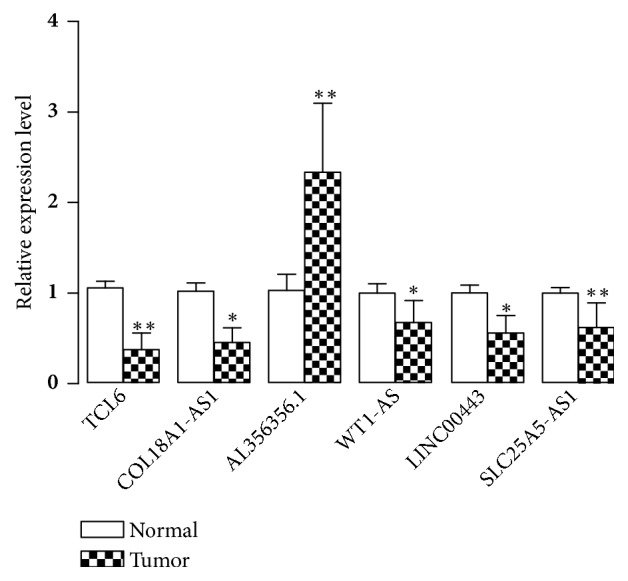
Expressions of* COL18A1-AS1, WT1-AS, LINC00443, TCL6, AL356356.1, *and* SLC25A5-AS1* were evaluated by RT-qPCR in tumor tissues and corresponding adjacent normal samples. Data presented as mean ± SE (*n* = 15 per group). ^*∗*^ *P* < 0.05 versus control; ^*∗∗*^ *P* < 0.001 versus control.

In terms of the above analysis from microarray analysis, these 6 lncRNAs (*COL18A1-AS1, WT1-AS, LINC00443, TCL6, AL356356.1, and SLC25A5-AS1*) involving ceRNA network were regarded as potential biomarkers linked with ccRCC patients (Table 4). To verify the validity and reliability of the results, the 6 lncRNAs were selected to analyze their expression between 15 diagnosed ccRCC specimens and 15 adjacent nontumor samples through qRT-PCR. As indicated in Figure 5, the results of qRT-PCR were consistent with our findings in computer analysis, implying that these lncRNAs may be acted as biomarkers in the process of ccRCC deterioration.

## 4. Discussion

RCC, a heterogeneous kind of tumors, mainly originates in renal tubular epithelial cells, more common in males after the age of 60 [1]. It has been documented that ccRCC as the most common pathological subtype of RCC is still leading to a high number of mortality in urologic neoplasms [24]. Therefore, in order to improve this situation, it is important to elucidate the ccRCC related genes and underlying molecular mechanisms involving in the progression of ccRCC.

Nowadays, increasing studies have showed the crucial biological functions of lncRNAs involved in the development and process of human diseases by epigenetic regulation and transcription as well as posttranscription regulation. A growing number of evidences have revealed that lncRNAs harbored similar sequences to their targeted miRNAs to regulate mRNAs expression in many tumor [25–27]. LncRNA ROR promotes radioresistance through acting as a ceRNA for microRNA145 to regulate RAD18 expression in hepatocellular carcinoma. LncRNA Gas5 serves as a ceRNA to activate PTEN/AKT pathway via sponging miR-222-3p in papillary thyroid carcinoma. Moreover, lncRNA PTENP1 could interact with miR-19b, upregulate the expression of p53, and downregulate p-AKT expression, inhibiting breast cancer cell growth and metastasis.

However, few researches between lncRNA and ccRCC have been achieved based on the large numbers of samples. In this regard, we comprehensively integrated 611 samples lncRNA, miRNA, and mRNA data from TCGA and investigated the lncRNAs related ceRNA network to uncover the underlying mechanism about ccRCC. In the present research, 34 DElncRNAs were identified in ccRCC samples compared with the normal samples. 6 out of them involved in the ceRNA network were significantly linked with overall survival might be considered as prognostic markers for ccRCC. Notably, two DElncRNAs, lncRNA WT1-AS and TCL6, interacted with most of the DEmiRNAs in ceRNA network. LncRNA WT1-AS, Wilms tumor 1 (WT1) gene antisense, locates in the upstream of WT1, which was originally found on the chromosome 11p and function as regulating in the development of paediatric kidney tumor originated from embryonal blastemal stem cells [28]. Kaneuchi* et al.* [29] found that the expression of WT1-AS was in parallel with WT1 gene in ovarian clear cell adenocarcinoma with poor prognosis. Moreover, WT1-AS could upregulate expression of WT1 to inhibit hepatocellular carcinoma cell proliferation and apoptosis via activating JAK2/STAT3 and MAPK signaling pathway [30]. Several studies have demonstrated that WT1-AS could be acted as a therapeutic target in the acute myeloid leukemia and gastric cancer as well as colon cancer [28, 31, 32]. Consistent with the researches above, lncRNA WT1-AS was also found aberrant expression in the 530 ccRCC samples compared with normal sample and the results of univariate and multivariate regression analysis showed that lncRNA WT1-AS could be acted as an independent predictor of ccRCC patients. Moreover, high expression of lncRNA WT1-AS was related to poor prognosis based on KM curve. Of interest is the fact that the results of RT-qPCR were in agreement with the bioinformatic analysis, confirming that this lncRNA could play an important role in the process of ccRCC. In addition, the result of ceRNA network showed that the targeted miRNAs of lncRNA WT1-AS were miR-141 miR-155 and miR-216b, suggesting that it may be acted as a sponge to compete with these three key DEmiRNAs participated in the pathogenesis mechanism of ccRCC carcinogenesis.

Another lncRNA TCL6, named T-cell leukemia/lymphoma 6 (TCL6), from a region on chromosome 14q locus was found in T-cell leukemia. The* TCL6 *gene could encode different open reading frames with no homology via through alternative splicing, leading to T-cell lymphoproliferative diseases [33]. Previous study has showed that overexpressed of TCL6 could suppress the proliferation and growth of ccRCC cells and the expression of TCL6 was negatively linked with TNM stage of tumor [34], which was similar to our results. The samples in our research were much more than their study with 71 tumor samples, Moreover, this variable in our univariate and multivariate regression analysis were both significantly lncRNAs with* P* value less than 0.05, and the results of ROC curve confirmed that lncRNA TCL6 could be considered as an independent predictor for ccRCC patients. However, they proved the hypothesis via some cellular functional experiments instead of further mechanism discussion. Our results showed that lncRNA TCL6 could be served as a ceRNA to in regulation the expression of targeted genes by sponging miR-210, miR-216b, and miR-122, further reinforcing their conclusion. Furthermore, other lncRNAs (COL18A1-AS1, LINC00443, AL356356.1, and SLC25A5-AS1) were also significantly aberrantly expressed in the ccRCC tumors compared with the normal samples, but there are scarcely reports about them until now. Therefore, further investigations are needed to identify the function and exact mechanism of these for ccRCC.

MiRNA still warrants to be considered as a crucial modulator in the regulation of tumors although lncRNA has obtained lots of attentions in these years. There is no doubt that targeted genes could be changed via interrupting miRNAs, resulting the aggressive tumorigenic state. In our research, the intersection results between two important lncRNA WT1-AS and TCL6 targeted miRNA involved ceRNA network showed that miR-216b played an important role for ccRCC. MiR-216b was first discovered to function as a tumor suppressor involving in regulating nasopharyngeal cell proliferation and invasion as well as tumor growth via inhibiting the KRAS/AKT and ERK pathways [35]. Subsequently, several researches further proved that miR-216b played significant in various tumors such as breast cancer, colorectal cancer, and hepatocellular carcinoma as well as pancreatic ductal adenocarcinoma [36–39]. Consistent with previous researches, our results also showed that the expression of miR-216b in tumor samples was abnormal and it participated in the ceRNA network for ccRCC. In addition, the targeted genes based on the online databases analysis mainly involved in metabolic pathway and cytokine-cytokine receptor interaction, and the most important functional alterations were sodium ion transport and extracellular region part, as well as sodium ion binding. These findings suggesting that miR-216b might influence targeted genes via sodium ion transporting from extracellular position in ccRCC carcinogenesis.

Nevertheless, our study has several limitations. Due to all the samples and patient clinical information in our study were based on TCGA, another independent database includes a large-scale multicenter clinical samples should be further verified our findings. In addition, the mechanism of ceRNA network suggests that one lncRNA can connect with numbers of miRNAs and relevant targeted mRNAs [11]. Nevertheless, the miRNAs and targeted mRNAs involved in ceRNA network were based on TCGA database in our research. In this regard, our further research will focus on validating these miRNAs and mRNAs and uncover the mechanism of ceRNA in vivo and in vitro to prove our discovery, especially to confirm the crucial interactions we discussed above.

In conclusion, dysregulated 34 cancer-specific lncRNAs were screened between tumor and nontumor samples from TCGA database. Moreover, we successfully constructed lncRNA-associated ceRNA network by integrating aberrant lncRNA, miRNA, and mRNA expression profiles. In addition, six lncRNAs were identified to be remarkably correlative with overall survival of patients and could be considered as candidate predictors. Obviously, our findings will contribute to further understanding of ccRCC pathogenesis and bring to light a novel ceRNA regulatory mechanism in ccRCC.

## Figures and Tables

**Table 1:** Primers used in RT-qPCR.

Gene symbol	Forward Primer 5′-3′	Reverse Primer 5′-3′
TCL6	CTATCCATTCAGCATCAGAGA	CACATACTCACGCATCCTT
COL18A1-AS1	CCAAGGTTGTGAGAAGATTC	CAGGAGCATTATTCAGCATT
AL356356.1	TGTGAGCCAAGAAGCATT	CTTACAGCCAGCATTCCA
WT1-AS	TGTTCTGAGGATTAGATAGGAG	GTATCAACGCCACATGGT
LINC00443	CAGAAGGTTGCTGTTCATTAC	CTCTTGGTCTTGAATAGGTGAT
SLC25A5-AS1	GAGGGCTTTATTTGGAGAGAGG	CAAGTGATGGCGAGGTGTATC
GAPDH	ATGACATCAAGAAGGTGGTG	CATACCAGGAAATGAGCTTG

**Table 2:** Gene ontology analysis of targeted genes in competitive endogenous RNA crosstalk associated with ccRCC.

Category	Term	Count	* P* Value
GOTERM_BP_FAT	GO:0006814_sodium ion transport	9	1.13E-04
GOTERM_BP_FAT	GO:0007267_cell-cell signaling	19	1.49E-04
GOTERM_BP_FAT	GO:0006811_ion transport	21	4.11E-04
GOTERM_BP_FAT	GO:0007155_cell adhesion	19	9.40E-04
GOTERM_BP_FAT	GO:0022610_biological adhesion	19	9.55E-04
GOTERM_BP_FAT	GO:0001568_blood vessel development	10	0.001881
GOTERM_BP_FAT	GO:0001944_vasculature development	10	0.002216
GOTERM_BP_FAT	GO:0035295_tube development	9	0.003551
GOTERM_BP_FAT	GO:0048562_embryonic organ morphogenesis	7	0.004006
GOTERM_BP_FAT	GO:0016337_cell-cell adhesion	10	0.004163
GOTERM_CC_FAT	GO:0044421_extracellular region part	29	1.66E-06
GOTERM_CC_FAT	GO:0005886_plasma membrane	68	4.69E-06
GOTERM_CC_FAT	GO:0044459_plasma membrane part	47	5.87E-06
GOTERM_CC_FAT	GO:0005615_extracellular space	21	6.13E-05
GOTERM_CC_FAT	GO:0016021_integral to membrane	82	8.52E-05
GOTERM_CC_FAT	GO:0031224_intrinsic to membrane	84	9.20E-05
GOTERM_CC_FAT	GO:0005576_extracellular region	40	2.03E-04
GOTERM_CC_FAT	GO:0001518_voltage-gated sodium channel complex	4	2.69E-04
GOTERM_CC_FAT	GO:0034706_sodium channel complex	4	5.43E-04
GOTERM_CC_FAT	GO:0000267_cell fraction	25	7.34E-04
GOTERM_MF_FAT	GO:0031402_sodium ion binding	9	4.42E-05
GOTERM_MF_FAT	GO:0005248_voltage-gated sodium channel activity	4	5.40E-04
GOTERM_MF_FAT	GO:0031420_alkali metal ion binding	10	9.44E-04
GOTERM_MF_FAT	GO:0005272_sodium channel activity	4	0.005137
GOTERM_MF_FAT	GO:0019838_growth factor binding	6	0.006049
GOTERM_MF_FAT	GO:0046983_protein dimerization activity	14	0.006839
GOTERM_MF_FAT	GO:0015293_symporter activity	6	0.017732

**Table 3:** Univariate and multivariate Cox regression analysis of six significantly lncRNAs associated with overall survival in TCGA ccRCC data set.

	Univariate regression model			Multivariate regression model		
	HR	95% CI	*P*-value	HR	95% CI	*P*-value
Test set (n = 530)						
COL18A1-AS1	0.748088	0.678-0.826	8.54E-09	0.5666	0.421-0.763	0.00018
WT1-AS	1.211644	1.131-1.30	4.41E-08	1.3891	1.134-1.702	0.00151
LINC00443	0.735709	0.627-0.864	0.000174	0.6937	0.553-0.870	0.00153
TCL6	0.853173	0.801-0.905	1.09E-07	0.7117	0.562-0.901	0.00463
AL356356.1	1.295632	1.145-1.466	3.93E-05	1.7526	1.045-2.934	0.03338
SLC25A5-AS1	0.824427	0.706-0.963	0.014589	4.6823	1.068-2.534	0.04069

**Table 4:** The detailed information of 6 aberrantly expressed lncRNAs in the ccRCC.

Ensemble ID	Gene symbol	FDR	Fold change	Location
ENSG00000187621	TCL6	3.66E-33	-2.77992	Chr 14: 95,650,498-95,679,833
ENSG00000183535	COL18A1-AS1	1.85E-65	-2.67464	Chr 21: 45,419,716-45,425,070
ENSG00000237781	AL356356.1	4.78E-29	2.15702	Chr 1: 150,548,562-150,557,724
ENSG00000183242	WT1-AS	4.91E-38	-2.88135	Chr 11: 32,435,518-32,458,769
ENSG00000230156	LINC00443	2.95E-118	-3.34318	Chr 13: 106,653,916-106,672,163
ENSG00000224281	SLC25A5-AS1	9.28E-139	-2.15088	Chr X: 119,466,034-119,469,098

Chr, chromosome; FDR, false discovery rate.

## Data Availability

The data used to support the findings of this study are available from the corresponding author upon request.

## Supplementary Materials

Supplementary 1
** Figure S1**: Venn diagrams of differential expressed mRNAs interacted with putative targeted mRNAs predicted by databases of TargetScan (v7.1), miRDB.

Supplementary 2
** Figure S2**: Kaplan-Meier (KM) analyses on 34 significantly lncRNAs with *P* value less than 0.05. Survival curves for patients in two groups, solid red lines represented high expression and green lines denoted low expression of each lncRNA.
