# Supplementary figures and images for: Identification of Six Potentially Long Noncoding RNAs as Biomarkers Involved Competitive Endogenous RNA in Clear Cell Renal Cell Carcinoma
Source: Biomed Res Int. 2018 Oct 11;2018:9303486. doi: 10.1155/2018/9303486 (PMC6201332; doi:10.1155/2018/9303486)

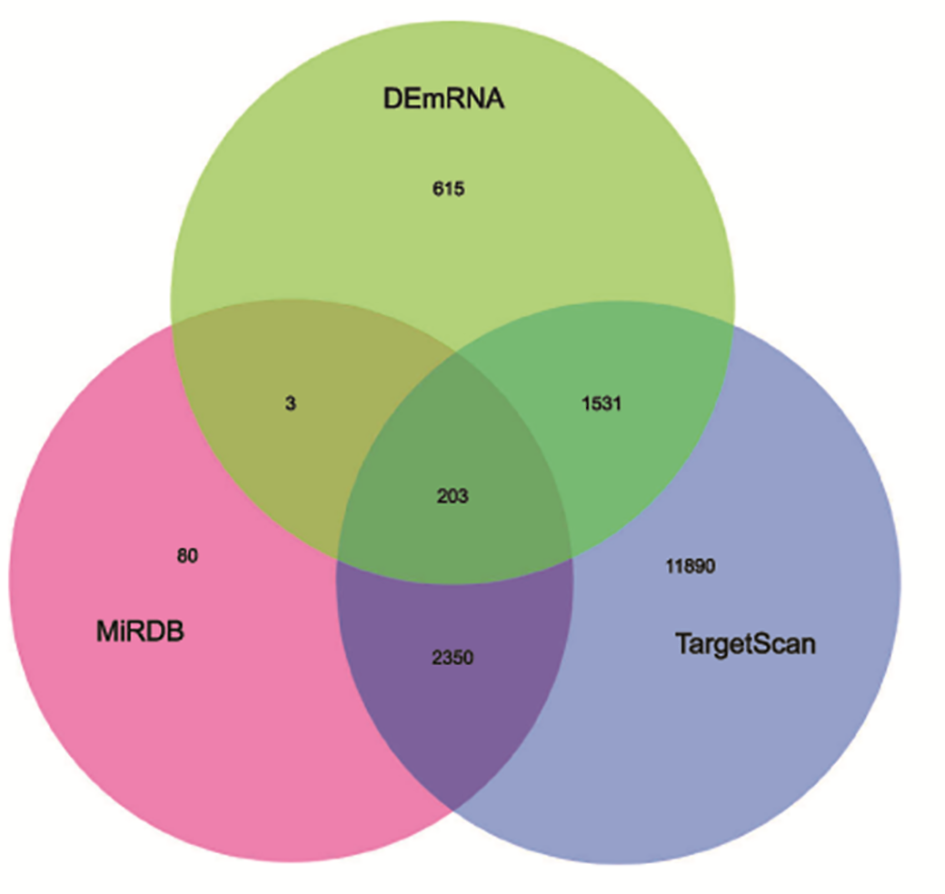

Supplement: Supplementary 1 — Figure S1: Venn diagrams of differential expressed mRNAs interacted with putative targeted mRNAs predicted by databases of TargetScan (v7.1), miRDB. [file 9303486.f1.tif]

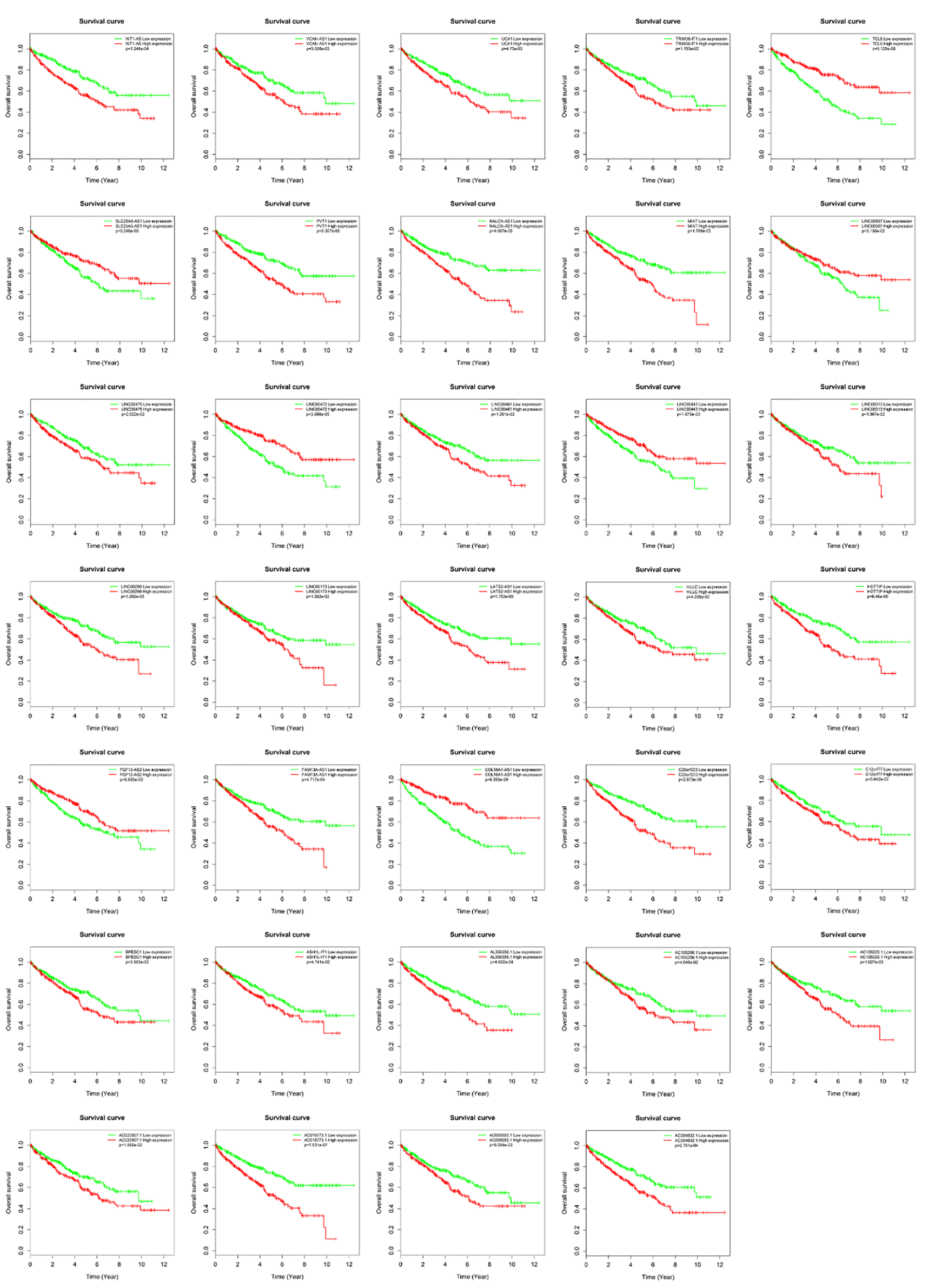

Supplement: Supplementary 2 — Figure S2: Kaplan-Meier (KM) analyses on 34 significantly lncRNAs with P value less than 0.05. Survival curves for patients in two groups, solid red lines represented high expression and green lines denoted low expression of each lncRNA. [file 9303486.f2.tif]
